# A Vaccine Strategy Based on the Identification of an Annular Ganglioside Binding Motif in Monkeypox Virus Protein E8L

**DOI:** 10.3390/v14112531

**Published:** 2022-11-16

**Authors:** Jacques Fantini, Henri Chahinian, Nouara Yahi

**Affiliations:** INSERM UMR_S-1072 and Faculty of Medicine, Aix-Marseille Université, Boulevard Pierre Dramard, 13015 Marseille, France

**Keywords:** pox virus, Monkeypox virus, vaccine, ganglioside, lipid raft

## Abstract

The recent outbreak of Monkeypox virus requires the development of a vaccine specifically directed against this virus as quickly as possible. We propose here a new strategy based on a two-step analysis combining (i) the search for binding domains of viral proteins to gangliosides present in lipid rafts of host cells, and (ii) B epitope predictions. Based on previous studies of HIV and SARS-CoV-2 proteins, we show that the Monkeypox virus cell surface-binding protein E8L possesses a ganglioside-binding motif consisting of several subsites forming a ring structure. The binding of the E8L protein to a cluster of gangliosides GM1 mimicking a lipid raft domain is driven by both shape and electrostatic surface potential complementarities. An induced-fit mechanism unmasks selected amino acid side chains of the motif without significantly affecting the secondary structure of the protein. The ganglioside-binding motif overlaps three potential linear B epitopes that are well exposed on the unbound E8L surface that faces the host cell membrane. This situation is ideal for generating neutralizing antibodies. We thus suggest using these three sequences derived from the E8L protein as immunogens in a vaccine formulation (recombinant protein, synthetic peptides or genetically based) specific for Monkeypox virus. This lipid raft/ganglioside-based strategy could be used for developing therapeutic and vaccine responses to future virus outbreaks, in parallel to existing solutions.

## 1. Introduction

Recently, an unexpected outbreak of the Monkeypox virus alerted national health agencies in Europe and North America [1]. Indeed, the number of transmission cases recorded in recent weeks greatly exceeds the usual periodic outbreaks of this virus outside Africa. In its update of 22 September 2022 [2], the Centers for Disease Control and Prevention (CDC) recommended the use of two vaccines for the prevention of Monkeypox and smallpox viruses: JYNNEOS, a replication-deficient vaccinia virus vaccine [3], and ACAM2000, a cell culture-based live vaccinia smallpox vaccine [4]. However, the effectiveness of such vaccines against a global epidemic of Monkeypox virus is not known. Indeed, in its September 2022 update, the CDC acknowledged the lack of consolidated data on the clinical efficacy or effectiveness of JYNNEOS or ACAM2000 for Monkeypox disease [2]. Due to these limitations in our knowledge about the effectiveness of these vaccines in the current outbreak, the CDC recommended that vaccinated people should continue to avoid close, skin-to-skin contact with monkeypox infected individuals. On the other hand, several drugs such as tecovirimat, cidofovir, brincidofovir, or vaccinia immunoglobulin could be used, but the risk-benefit balance of such treatments for Monkeypox infection is unclear [5]. Under these conditions, any effort to quickly develop alternative prevention strategies that can limit the spread of this virus is welcome.

To trigger an immune response acting at the earliest stages of the infection, it is first necessary to identify the proteins of the virus responsible for its adhesion to the host cell surface, as those proteins may be used as antigens in a vaccine formulation. In the case of Monkeypox virus, the cell surface-binding protein E8L is an interesting target, as it plays a critical role in viral entry [6].

An important feature of pox viruses is that the mature viral particles bind to polyanionic compounds such as glycosaminoglycans [7]. Since these cellular components are electronegatively charged at physiological pH, this means that the viral cell surface-binding protein must be electropositive [8]. Thus, like most enveloped viruses, pox viruses may select electronegative areas as primary landing platforms on host cell membranes. For this reason, lipid rafts, which are enriched in negatively charged gangliosides [9], are routinely used by most viruses to gain entry into host cells [10,11,12,13,14,15,16,17], and this is also the case for pox viruses [18,19,20]. Incidentally, ganglioside-binding domains of viral proteins are privileged targets for antiviral drugs and neutralizing antibodies, as demonstrated for a broad range of viruses [21], including HIV [22,23] and SARS-CoV-2 [8,10,12,24,25,26,27]. In the present study, we identified an annular ganglioside-binding motif on the Monkeypox virus E8L protein. This motif overlaps with three potential B linear epitopes which could be used in a safe and specific vaccine formulation against the Monkeypox virus.

## 2. Materials and Methods

### 2.1. Structure of the E8L Protein

The amino acid sequence of the Monkeypox virus E8L protein (Strain Zaire-96-I-16) was retrieved from the Uniprot entry Q8V4Y0 (Figure S1). The 304 amino acids of the sequence were submitted to the Robetta server (https://robetta.bakerlab.org, accessed on 1 September 2022) for 3D structure prediction [28,29]. Three topological domains were suggested by the Uniprot entry: virion surface (1–275), transmembrane (276–294) and intra-virion (295–304). 

### 2.2. Docking of E8L on Lipid Raft Gangliosides

In the 3D model predicted by the Robetta server, the 1–245 region formed a globular domain that was docked onto a preformed GM1 ganglioside cluster using a previously described molecular modeling strategy [30]. Several types of E8L-ganglioside complex were obtained after energy minimization with the Polak-Ribière algorithm of Hyperchem (Hypercube Inc., Gainesville, FL, USA), using the Charmm force field. We selected the complex with the highest energy of interaction as calculated by the Molegro Molecular Viewer software (Molexus, Odder, Denmark) as previously described [8]. This in silico strategy was developed by our group for studying the interaction of several SARS-CoV-2 variant spike proteins with host cell membranes and lipid raft gangliosides [24,31,32,33,34]. GM1 was chosen as a representative ganglioside because it is widely expressed on human cells, especially on mucosal tissues that serve as a common portal of entry for a broad range of viruses [10,16,35,36]. The total energies of binding of the E8L-raft complexes were calculated by the Ligand Energy Inspector Tool in software Molegro Molecular Viewer [37]. This tool allows getting detailed information about the energy interactions for each E8L-GM1 interaction (Figure S2).

### 2.3. Prediction of Linear B Epitopes

The prediction of linear B epitopes usually combines an analysis of the following physicochemical properties: hydrophilicity [38], flexibility [39], accessibility [40], turns [41], exposed surface [42], polarity [43], and antigenic propensity [44]. Several B epitope prediction servers are available online. For this study, we submitted the FASTA sequence of the E8L protein (Uniprot entry Q8V4Y0) to BepiPred-3.0 [45] (https://services.healthtech.dtu.dk/service.php?BepiPred-3.0, accessed on 9 November 2022) (Figure S3) and to BcePred [46] (https://webs.iiitd.edu.in/raghava/bcepred/, accessed on 1 October 2022) (Figure S4). The potential epitopes identified by these methods overlapped the predictions published by Shantier et al. [47] (epitopes 43–62, 94–113 and 204–223) which were analyzed in the present study.

## 3. Results

### 3.1. Structure of E8L in the Unbound and Ganglioside-Bound Configurations

**Figure 1:**
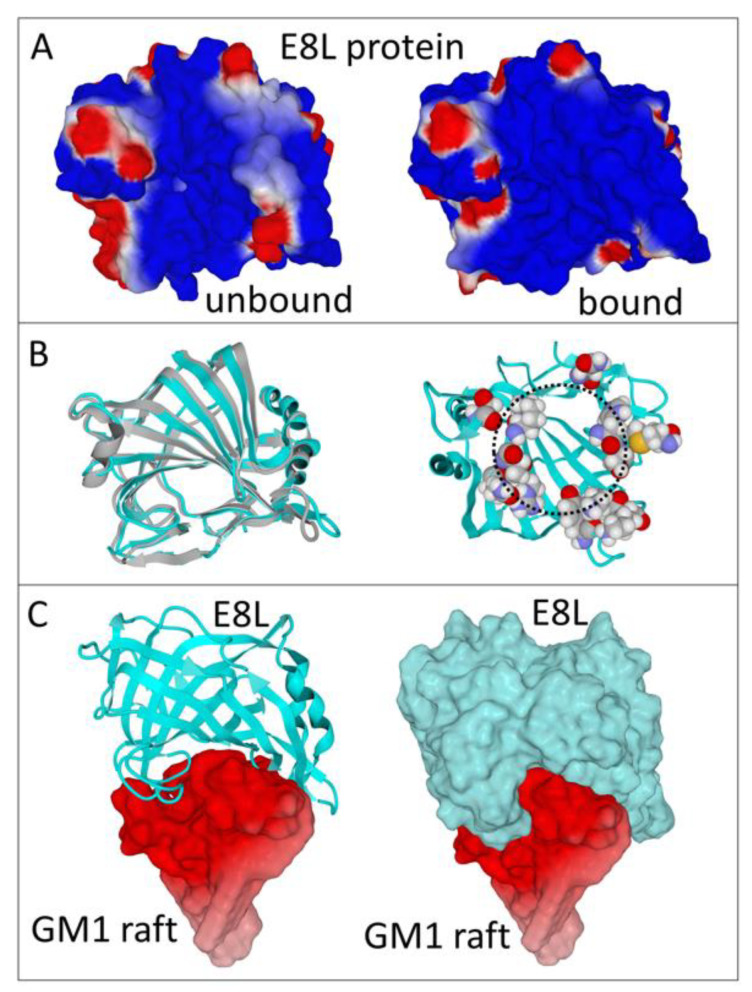
Identification of a ganglioside-binding site at the tip of the E8L monkeypox virus protein. (**A**) Electrostatic surface potential of the E8L protein side facing the host cell. Two conformations (unbound and bound to gangliosides) are shown. Electropositive, electronegative, and neutral regions are colored in blue, red, and white, respectively. Note the increase of electropositive surfaces on the bound protein. (**B**) Secondary structure organization of the E8L protein before (grey) and after binding to gangliosides (cyan). Both structures are superposed on the left panel. On the right panel, the annular distribution (dashed circle) of the amino acid residues involved in ganglioside binding are represented in atomic spheres (carbon in grey, oxygen in red, nitrogen in blue, hydrogen in white, sulfur in yellow). (**C**) E8L protein bound to a cluster of gangliosides GM1 in a typical lipid raft organization. On the left panel, the E8L protein is represented in ribbons. On the right panel, the protein is shown in surface rendering. In both cases the electrostatic surface potential of the GM1 raft is shown.

There is no available 3D structure of the E8L protein. So, we had to develop a molecular model of this protein from its amino acid sequence (Figure S1). The model we obtained is described in Figure 1. As we indicated in the Materials and Methods, we chose to focus our study on the 1–245 part, which corresponds to the globular surface-exposed domain of the E8L protein. The electrostatic potential of E8L is initially largely electropositive on the unbound protein, especially in the region that faces the plasma membrane of the host cell (Figure 1A, left panel). However, when the protein interacts with membrane gangliosides, a specific induced-fit process affects the balance between acidic and basic amino acid side chains, leading to a further increase of the electrostatic surface potential (Figure 1A right panel). These conformational changes have a limited impact on the secondary structure of the protein, as evidenced by the superposition of these structures before and after binding to gangliosides (Figure 1B, left panel). The essential structural feature of the ganglioside-binding motif of E8L is that it consists of several subsites forming a ring structure (Figure 1B, right panel). This annular structure allows a perfect adaptation of the respective shapes of the protein and of the ganglioside cluster (Figure 1C). Remarkably, the protein has a cavity in its center, whose limits are constituted by the ring pattern. When the complex is formed, this cavity literally covers the ganglioside cluster and wraps the lipid raft.

**Figure 2:**
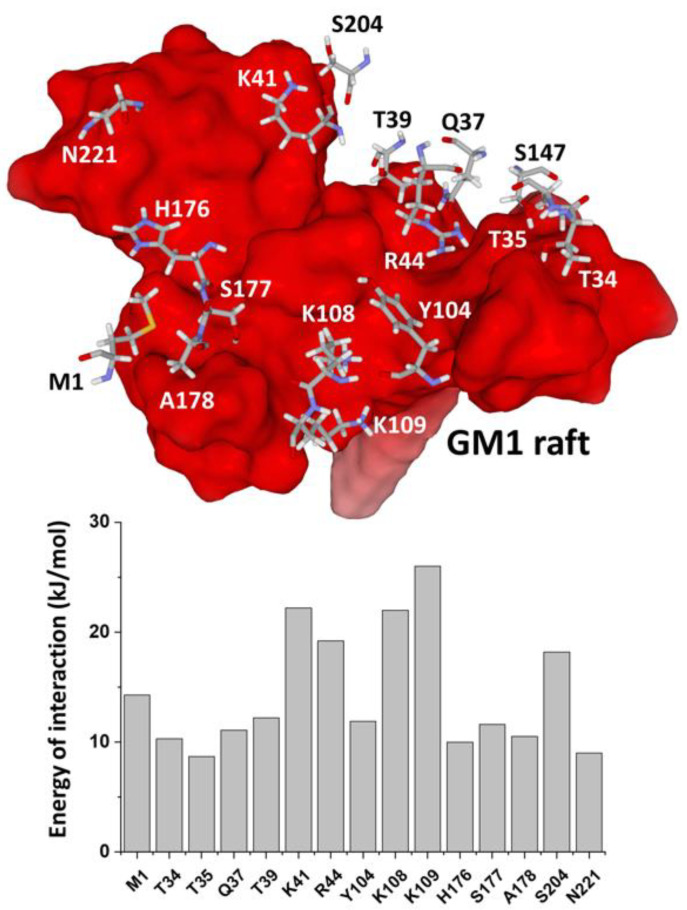
Molecular structure of the ganglioside-binding motif of the E8L protein. On the upper panel, the main amino acid residues involved in ganglioside binding (carbon in grey, oxygen in red, nitrogen in blue, hydrogen in white, sulfur in yellow) are represented over the GM1 cluster (electrostatic potential mostly negative represented in red). On the lower panel, the histograms show the contribution of each of these amino acids to the total energy of interaction (∆G in kJ.mol^−1^) of the E8L-ganglioside complex.

The energy of interaction of the E8L-GM1 complex is estimated at −350 kJ.mol^−1^. This value is similar to the one obtained for the SARS-CoV-2 spike protein N-terminal domain (NTD) bound to a similar GM1 raft [8]. We identified 15 key amino acid residues (Figure 2, upper panel) that account for 63% of the total energy of interaction (Figure S2). Among those amino acids, lysine and arginine residues are the most significant contributors, which is expected given their positive charge (Figure 2, lower panel): K41, R44, K108 and K109. Interestingly, the ganglioside-binding motif does not include any cluster of acidic amino acid residues, which is consistent with the large electropositive protein surface that faces the host cell membrane (Figure 1A). The global increase of the electrostatic surface potential of E8L upon ganglioside binding is perfectly illustrated by the characteristic folding of K41 (Figure 2, upper panel). The side chain of this basic amino acid residue folds on itself to minimize the surface occupied by the apolar groups, which leads to a better surface exposure of the positive charge carried by the terminal ε-NH_3_^+^ group.

### 3.2. Identification of Surface-Exposed Linear B Epitopes in E8L

**Figure 3:**
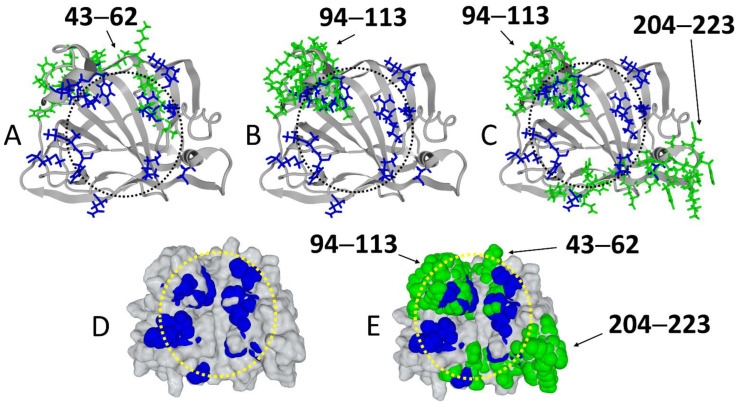
Predicted B epitopes overlap the annular ganglioside-binding motif of the E8L protein. The amino acid residues that constitute the three potential B epitopes are colored in green. These epitopes lie within the 43–62 (**A**), 94–113 (**B**) and 204–223 (**C**) segments of the E8L amino acid sequence. Note that the 94–113 and 204–223 are separate epitopes (**C**) whereas 43–62 and 94–113 significantly overlap (**A**,**B**). In the unbound E8L protein, some amino acid residues belonging to the ganglioside-binding domain (blue spheres) are not immediately accessible on the protein surface (**D**). However, the three epitopes (represented in green atomic spheres) are fully accessible (**E**). The dashed circles underscore the annular organization of the ganglioside-binding motif.

Then we searched for potential linear B epitopes in the amino acid sequence of the E8L protein (Figure 3). In agreement with a recent study [47], we identified three distinct amino acid segments that are consensually predicted to induce the production of antibodies: 43–62 (VRINFKGGYISGGFLPNEYV), 94–113 (VHWNKKKYSSYEEAKKHDDG), and 204–223 (SSSNHEGKPHYITENYRNPY) (Figure 3A–C, Figures S1, S3 and S4).

The 43–62 epitope spans two ganglioside-binding domain subsites that represent about half of the entire motif (Figure 3A). Epitope 94–113 partially overlaps one of these two subsites (Figure 3B). As for epitope 204–223, it spans two subsites, but it does not overlap with any of the other two epitopes. Analysis of the ganglioside-binding domain at the level of the unbound form of the E8L protein highlights the fact that some of the amino acids of the motif are not directly accessible (Figure 3D), in agreement with the data in Figure 1A. Moreover, our modeling studies suggest that the three epitopes 43–62, 94–113, and 204–223 are exposed on the surface of the protein in its unbound configuration. This localization is clearly visible when the amino acids of the ganglioside-binding domain are superimposed on those of the three epitopes (Figure 3E). Thus, antibodies raised against these epitopes of the E8L protein are expected to neutralize infection by preventing the binding of the Monkeypox virus to the host cell membrane.

## 4. Discussion

In the last years, our team developed a paradigm according to which the structural dynamics of enveloped viruses is largely based on the acquisition, maintenance, and reinforcement of the surface electrostatic potential [8,9]. We have developed unique molecular modeling approaches that have successfully predicted protein-ganglioside interactions in various research fields, including neurobiology [48] and microbiology [9]. Indeed, we have correctly anticipated the immune escape of the first omicron variant of SARS-CoV-2 [30]. We have also given mechanistic explanations accounting for the respective advantages of each SARS-CoV-2 variant by studying the surface electrostatic potential of the NTD domain of their spike protein, in relation to the kinetics of viral adhesion to lipid rafts [8]. In this respect, basic amino acids (lysine and arginine) allow cell surface-binding proteins to interact optimally with lipid rafts, which are negatively charged areas of the plasma membrane of host cells, due to their natural enrichment in gangliosides [48]. For this reason, neutralizing antibodies are often directed against the domains of viral proteins that interact with lipid rafts [24]. In this respect, it is of primary importance to identify functional ganglioside-binding domains in cell surface-binding proteins expressed on viral envelopes. In silico approaches have been successfully used in the last years to detect such binding motifs in microbial proteins and toxins, which now form a family of three distinct topologies including helices, loops, and large flat surfaces [9]. Interestingly, the Monkeypox E8L protein adds a new category, i.e., a ring-like annular structure that forms the wall of a small hollow cylinder which sits on top of the raft, at the limited cost of a fine-tuning consisting in the repositioning of selected acidic and basic amino acids. Further studies focused on surface proteins from different viruses will allow it to be determined whether this new topology is specific to Monkeypox virus or if it is more generally encountered. It will also be interesting to study the kinetics of viral protein-binding to ganglioside clusters and determine how much of an induced-fit mechanism is needed for each virus.

In the present study, our strategy was to combine (i) the search for such ganglioside recognition sites on the Monkeypox virus E8L protein, and (ii) a B epitope prediction strategy. B-cell epitopes are particularly interesting to consider for designing peptide-based vaccines [49], which in the case of viruses may be desirable for obtaining only neutralizing antibodies without the risk of antibody enhancement of infection (ADE) [31]. In this regard, the identification of antigenic sequences important for the development of rapid and safe synthetic vaccines is crucial in these pandemic days [50]. Thanks to this multiparametric approach, among the potential epitopes predicted by the BepiPred (Figure S3) and the BcePred (Figure S4) servers, we have identified which ones are both surface-expressed and located on the right side of the E8L protein, i.e., on the side facing the host cell. Both properties are mandatory if one wants to generate neutralizing rather than facilitating antibodies [10,31]. This strategy allowed us to select three linear epitopes encompassing amino acids 43–62, 94–113, and 204–223 that overlap with the annular ganglioside-binding motif of E8L. All these epitopes belong to E8L regions that are well conserved among Monkeypox virus sequences analyzed so far [47]. We can therefore suggest using these three sequences derived from the E8L protein as immunogens in a vaccine formulation (recombinant protein, synthetic peptides or genetically based) specific for Monkeypox virus. The recombinant protein could be the extra-viral domain 1–245. Synthetic peptides could include either individual linear epitopes 43–62, 94–113, and 204–223 (or a part of these epitopes), alone or in combination. In the latter case, the 204–223 epitope could be mixed with either 43–62 or 94–113. However, 43–62 and 94–113 significantly overlap on the 3D structure of the E8L protein and thus should not be combined in a vaccine formulation. Finally, a genetically-based formulation coding for the E8L protein (preferentially the 1–245 domain), such as a mRNA-delivered lipid particle [51] also warrants consideration. We hope that these suggestions, which are not the subject of any patent filing, will be used by academic laboratories and/or pharmaceutical companies in the context of the global fight against the monkeypox virus. If we put in parallel (i) the regions of virus surface envelope proteins controlling the interaction with lipid raft gangliosides and (ii) the identification of linear B epitopes belonging to these regions, we could develop an innovative approach to quickly test vaccine solutions in the event of a new viral pandemic. This strategy could prove to be particularly useful in the event of the sudden emergence of a new variant of a known virus or in the event of the transmission to humans of new viruses for which no vaccine exists. In this regard, a recent study showed that simian hemorrhagic fever virus (SHFV, an arterivirus) is able to infect human cells, suggesting a potential risk of zoonotic transmission [52]. Since lipid rafts are required in arteriviruses infection [53], the approach described in the present study could also be used in the case of SHFV spillover.

## Data Availability

Not applicable.

## Supplementary Materials

The following supporting information can be downloaded at: https://www.mdpi.com/article/10.3390/v14112531/s1, Figure S1: Amino acid sequence of Monkeypox E8L protein (Uniprot entry Q8V4Y0); Figure S2: Extraction of energies of interaction of the E8L-raft complex from Molegro (raw data). Figure S3: B epitope prediction by BepiPred (raw data); Figure S4: B epitope prediction by BcePred (raw data).
