# Supplementary material for: A Vaccine Strategy Based on the Identification of an Annular Ganglioside Binding Motif in Monkeypox Virus Protein E8L
Source: Viruses. 2022 Nov 16;14(11):2531. doi: 10.3390/v14112531 (PMC9693861; doi:10.3390/v14112531)

Supplementary Materials

A Vaccine Strategy Based on the Identification of an Annular Ganglioside Binding Motif in Monkeypox Virus Protein E8L

Jacques Fantini [1]*, Henri Chahinian [1] and Nouara Yahi [1]

[1]INSERM UMR_S-1072 and Faculty of Medicine, Aix-Marseille Université, Boulevard Pierre Dramard 13015 Marseille, France
*Correspondence: jacques.fantini@univ-amu.fr

Figure S1: Amino acid sequence of Monkeypox E8L protein (Uniprot entry Q8V4Y0)

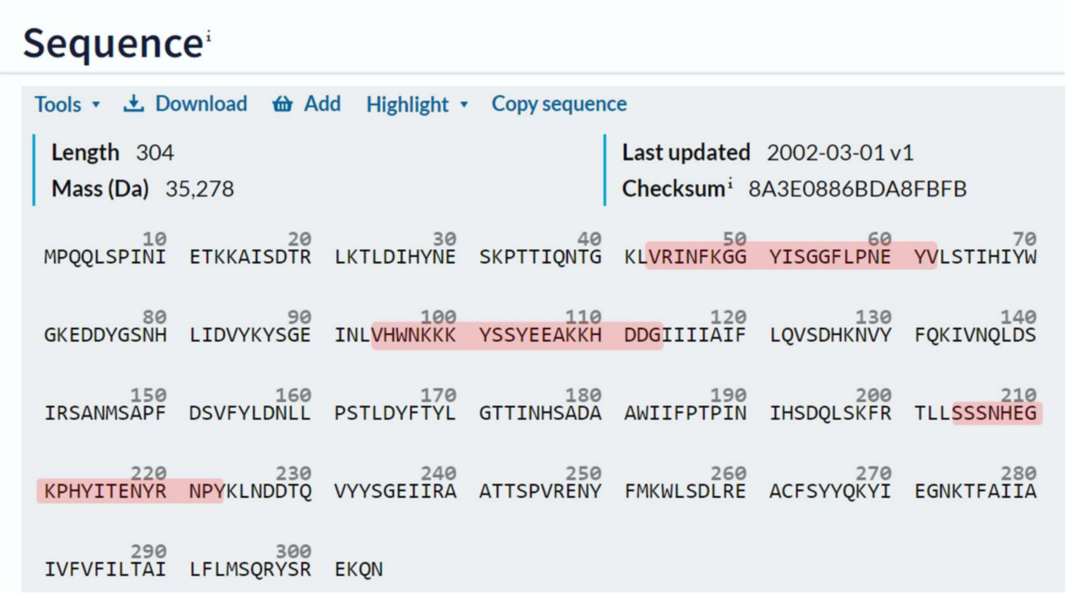

The three B linear epitopes are highlighted:

43–62 (VRINFKGGYISGGFLPNEYV)

94–113 (VHWNKKKYSSYEEAKKHDDG)

204–223 (SSSNHEGKPHYITENYRNPY)

Figure S2: Extraction of energies of interaction of the E8L-raft complex from Molegro (raw data)

Ligand Energy Inspector

Ligand/pose: GM1_1 [B] ☐ Hide other

Ligand | Targets | Total Energy | Settin

Show Residue / Molecule Contributions

Molecule	Residue	ID	Total
Noname [A]	Ala	144	-6.62817
Noname [A]	Arg	44	-19.9676
Noname [A]	Arg	220	-0.944819
Noname [A]	Asn	38	-0.794637
Noname [A]	Asn	46	-8.80164
Noname [A]	Asn	145	-7.23314
Noname [A]	Asn	175	-3.40928
Noname [A]	Asp	179	0.330276
Noname [A]	Gln	37	-12.9307
Noname [A]	Glu	105	-5.15282
Noname [A]	His	67	-1.95558
Noname [A]	His	176	-3.95049
Noname [A]	Ile	114	-0.38957
Noname [A]	Leu	42	-0.310442
Noname [A]	Lys	41	-0.610174
Noname [A]	Lys	108	-6.31572
Noname [A]	Lys	109	-0.505778
Noname [A]	Ser	147	-12.3345
Noname [A]	Ser	177	-1.94291
Noname [A]	Thr	35	-5.18735
Noname [A]	Thr	39	-13.3723
Noname [A]	Thr	173	-3.59116
Noname [A]	Tyr	104	-3.65703

Copy tables to clipboard

Ligand Energy Inspector

Ligand/pose: GM1_1 [C] ☐ Hide other

Ligand | Targets | Total Energy | Settin

Show Residue / Molecule Contributions

Molecule	Residue	ID	Total
Noname [A]	Arg	44	-0.988796
Noname [A]	Asn	46	-2.51014
Noname [A]	Asp	151	0.885167
Noname [A]	Glu	30	0.314451
Noname [A]	Leu	63	-0.715612
Noname [A]	Lys	32	-0.853838
Noname [A]	Lys	48	-6.42003
Noname [A]	Lys	108	-0.364192
Noname [A]	Lys	109	-0.336556
Noname [A]	Lys	133	-0.325698
Noname [A]	Phe	47	-5.39782
Noname [A]	Ser	64	-5.40563
Noname [A]	Ser	103	-3.45476
Noname [A]	Thr	34	-10.2736
Noname [A]	Thr	35	-4.31777
Noname [A]	Trp	96	-4.63924
Noname [A]	Tyr	104	-8.2449

Energies of interaction of E8L-GM1 complexes (6 GM1 gangliosides)
[A] = E8L
[B] to G [G] = GM1
Raw data extracted from Molegro

Ligand Energy Inspector

Ligand/pose: GM1_1 [D] ☐ Hide other

Ligand | Targets | Total Energy | Settin

Show Residue / Molecule Contributions

Molecule	Residue	ID	Total
Noname [A]	Arg	220	-5.11164
Noname [A]	Glu	217	1.04046
Noname [A]	Gly	40	-4.94305
Noname [A]	His	213	-5.27696
Noname [A]	Lys	41	-20.9463
Noname [A]	Lys	72	-0.605367
Noname [A]	Ser	204	-18.1525
Noname [A]	Ser	205	-6.51055
Noname [A]	Tyr	214	-0.421987

Ligand Energy Inspector

Ligand/pose: GM1_1 [E] ☐ Hide other

Ligand | Targets | Total Energy | Settin

Show Residue / Molecule Contributions

Molecule	Residue	ID	Total
Noname [A]	Asp	179	1.05052
Noname [A]	Glu	105	-2.30057
Noname [A]	Lys	108	-8.54293
Noname [A]	Lys	109	-3.28805

Ligand Energy Inspector

Ligand/pose: GM1_1 [F] ☐ Hide other

Ligand | Targets | Total Energy | Settin

Show Residue / Molecule Contributions

Molecule	Residue	ID	Total
Noname [A]	Arg	220	-1.66034
Noname [A]	Asn	221	-8.98143
Noname [A]	Glu	217	0.773193
Noname [A]	His	176	-0.30042
Noname [A]	Lys	41	-0.578257
Noname [A]	Lys	72	-0.665024
Noname [A]	Lys	86	-0.351114
Noname [A]	Tyr	223	-2.4494

Ligand Energy Inspector

Ligand/pose: GM1_1 [G] ☐ Hide other

Ligand | Targets | Total Energy | Settin

Show Residue / Molecule Contributions

Molecule	Residue	ID	Total
Noname [A]	Ala	178	-10.5365
Noname [A]	Asp	179	-8.67252
Noname [A]	Glu	105	0.857007
Noname [A]	His	110	-3.88199
Noname [A]	His	176	-6.21411
Noname [A]	Lys	108	-6.76304
Noname [A]	Lys	109	-21.8219
Noname [A]	Met	1	-14.2874
Noname [A]	Ser	177	-9.65025

Figure S3: B epitope prediction by BepiPred (raw data)

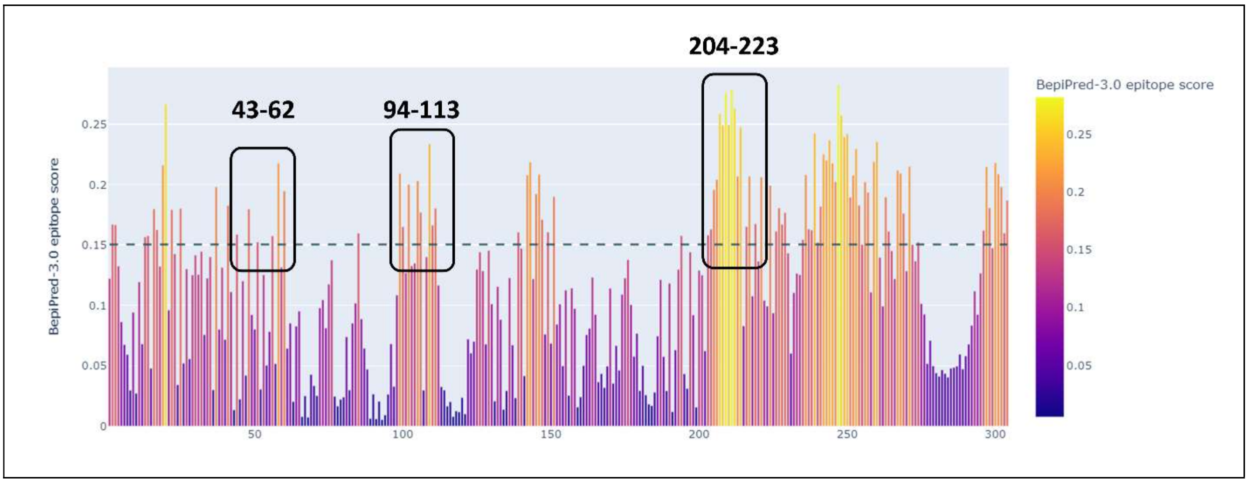

Figure S4: B epitope prediction by BcePred (raw data)

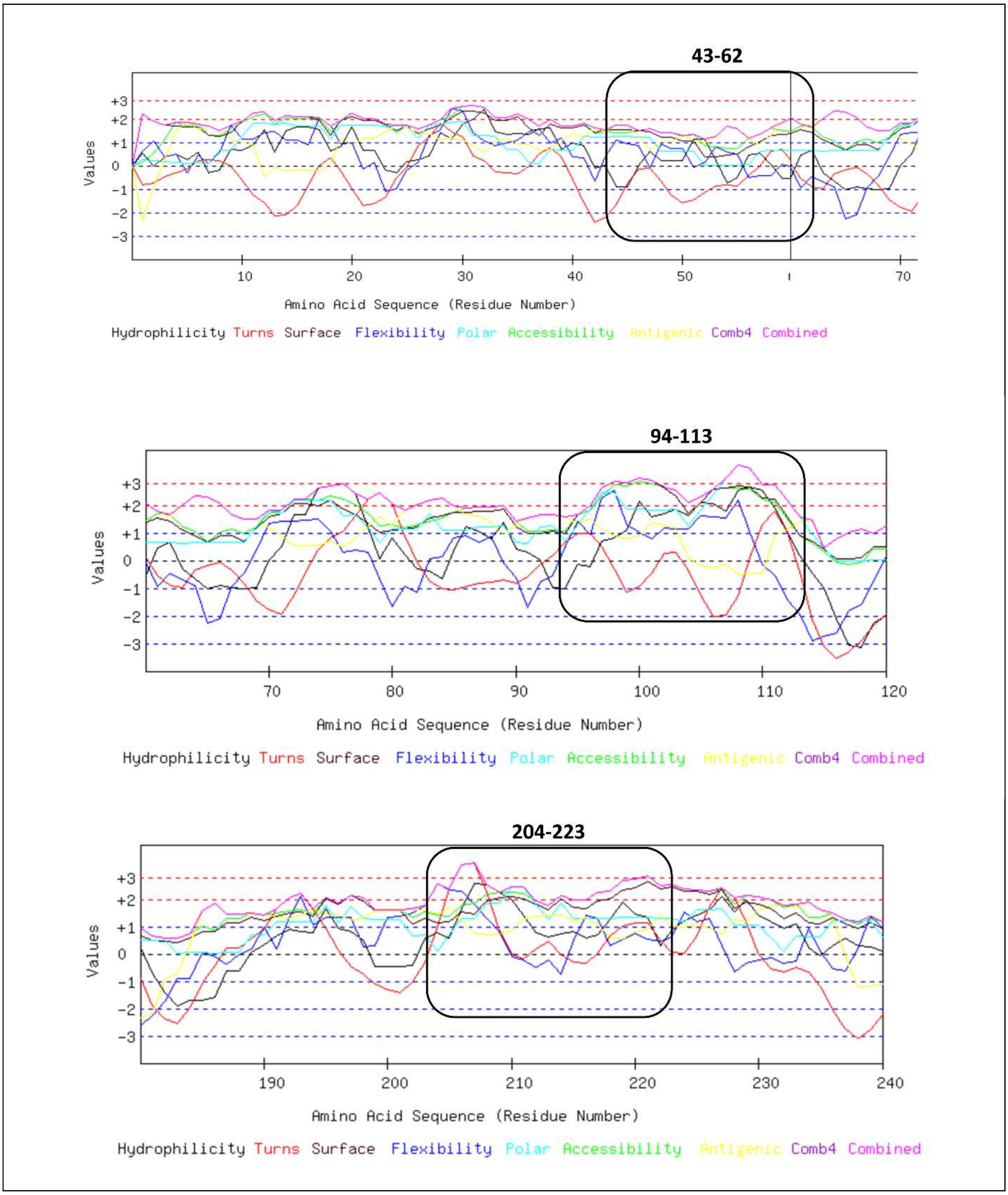

Supplement: Supplementary file 1 [file viruses-14-02531-s001.zip › viruses-1987044-supplementary.pdf]
